# An improved deep reinforcement learning routing technique for collision-free VANET

**DOI:** 10.1038/s41598-023-48956-y

**Published:** 2023-12-08

**Authors:** Pratima Upadhyay, Venkatadri Marriboina, Samta Jain Goyal, Sunil Kumar, El-Sayed M. El-Kenawy, Abdelhameed Ibrahim, Amel Ali Alhussan, Doaa Sami Khafaga

**Affiliations:** 1 Department of Computer Science and Engineering, Amity School of Engineering and Technology, Amity University Gwalior, Gwalior, Madhya-Pradesh India; 2 Deptartment of Computer Science and Engineering, SVKM’s NMIMS MPSTME Shirpur, Shirpur, India; 3 School of Computer Science, University of Petroleum and Energy Studies, Dehradun, India; 4 Department of Communications and Electronics, Delta Higher Institute of Engineering and Technology, Mansoura, 35111 Egypt; 5 Computer Engineering and Control Systems Department, Faculty of Engineering, Mansoura University, Mansoura, 35516 Egypt; 6 Department of Computer Sciences, College of Computer and Information Sciences, Princess Nourah bint Abdulrahman University, PO Box 84428, Riyadh, 11671 Saudi Arabia

**Keywords:** Computational science, Information technology

## Abstract

Vehicular Adhoc Networks (VANETs) is an emerging field that employs a wireless local area network (WLAN) characterized by an ad-hoc topology. Vehicular Ad Hoc Networks (VANETs) comprise diverse entities that are integrated to establish effective communication among themselves and with other associated services. Vehicular Ad Hoc Networks (VANETs) commonly encounter a range of obstacles, such as routing complexities and excessive control overhead. Nevertheless, the majority of these attempts were unsuccessful in delivering an integrated approach to address the challenges related to both routing and minimizing control overheads. The present study introduces an Improved Deep Reinforcement Learning (IDRL) approach for routing, with the aim of reducing the augmented control overhead. The IDRL routing technique that has been proposed aims to optimize the routing path while simultaneously reducing the convergence time in the context of dynamic vehicle density. The IDRL effectively monitors, analyzes, and predicts routing behavior by leveraging transmission capacity and vehicle data. As a result, the reduction of transmission delay is achieved by utilizing adjacent vehicles for the transportation of packets through Vehicle-to-Infrastructure (V2I) communication. The simulation outcomes were executed to assess the resilience and scalability of the model in delivering efficient routing and mitigating the amplified overheads concurrently. The method under consideration demonstrates a high level of efficacy in transmitting messages that are safeguarded through the utilization of vehicle-to-infrastructure (V2I) communication. The simulation results indicate that the IDRL routing approach, as proposed, presents a decrease in latency, an increase in packet delivery ratio, and an improvement in data reliability in comparison to other routing techniques currently available.

## Introduction

Vehicular Ad Hoc networks (VANETs) are comprised of vehicle units (VUs) that are strategically positioned in close proximity to road segments to facilitate communication. Wireless communication is utilized to establish a connection between vehicles or Road Side Units (RSUs) in close proximity^1, 2^. The users in VU obtain the desired services through internet connectivity^3^. The VU typically exhibits heightened mobility^4^, resulting in dynamic alterations to the topology of the network. Consequently, the linkage frequently exhibits instability. The limitations are additionally enforced by a vehicle’s interference and transmission range, as stated in reference^5^. The delay in routing is observed to escalate proportionally with the number of network hops that packets traverse during forwarding^6^, opting for road sections with high vehicle density can effectively mitigate delays. Typically, this factor is considered during protocol routing.

### VANET architecture

The ad hoc network infrastructures of Vehicular Ad-hoc Networks (VANETs) are experiencing a rapid expansion^7^, facilitated by wireless communication that interconnects vehicles. The utilization of VANETs has been observed as a recent development in the domain of transportation engineering, aimed at augmenting road safety, optimizing traffic flow, and mitigating traffic congestion and guidance issues^8^. The Onboard Units (OBUs) and Roadside Units (RSUs) facilitate effective communication between vehicles in both Vehicle-to-Vehicle (V2V) and Vehicle-to-Infrastructure (V2I) scenarios, as noted in reference^9^.

The wireless technology employed in Vehicular Ad-hoc Networks (VANETs), commonly referred to as Wireless Access in Vehicle Environment (WAVE), facilitates communication between vehicles and Roadside Units (RSUs)^10^. The WAVE communication system plays a crucial role in ensuring passenger safety by providing real-time traffic and vehicle information updates^11^. Further, imporoves the safety of both pedestrians and drivers, while also enhancing traffic flow and efficiency^12^. The classic model of Vehicular Ad-hoc Networks (VANETs) includes On-Board Units (OBUs), Traffic Analyzers (TAs), and Roadside Units (RSUs). The RSU serves as a platform for hosting applications that enable communication with other devices. Additionally, it aids the OBU in connecting to the Vehicle Unit (VU) to gather pertinent information regarding the vehicle, such as its speed, location, and fuel status. Subsequently, the aforementioned information is conveyed through the wireless network to the adjacent automobiles^13^. All roadside unit (RSU) interconnected with each other is also connected to TA via a wired network^14^. Furthermore, the TA assumes the duty of upholding authentication within Vehicular Ad-Hoc Networks (VANETs).

- **RSU** The Roadside Unit (RSU) is situated at a designated location, possesses a substantial storage capacity, and boasts formidable communication capabilities. Furthermore, it provides localized connectivity to vehicles traversing the road segments^15^. RSUs have the capability to establish communication with other network devices through wired links or wireless channels within the infrastructure networks^16^.
- **OBU** The vehilcle is outfitted with an On-Board Unit (OBU) that establishes wireless communication with either Roadside Units (RSU) or adjacent vehicles at regular intervals while ensuring confidentiality. OBU also uses input power, where each VU has a Global Positioning System (GPS) and reverse input sensors for the OBU^17^.
- **TA** Trusted authorities are responsible for the management of the VANET system, such as registration of vehicle users, RSUs and OBUs^18^. It is also responsible for ensuring the safety management of VANETs by validating the authentication of the vehicles in terms of user ID and OBU ID^19^. TA is operated with high power and large memory sizes to enable verification over entire vehicles and reveal the information of malicious message from a vehicle and it identifies the attackers^20^.

### Communication methods in VANETs

The Intelligent Transportation System (ITS) prioritizes the promotion of safe transportation and effective communication regarding road safety^21^. To address the issue of road congestion, ITS employs diverse networking techniques such as MANETs and VANETs^22, 23^. V2X communications are a crucial component of Intelligent Transportation Systems (ITS) as they enhance traffic management by providing dependable information on road bottlenecks and other transportation services^24^.

**Figure 1:**
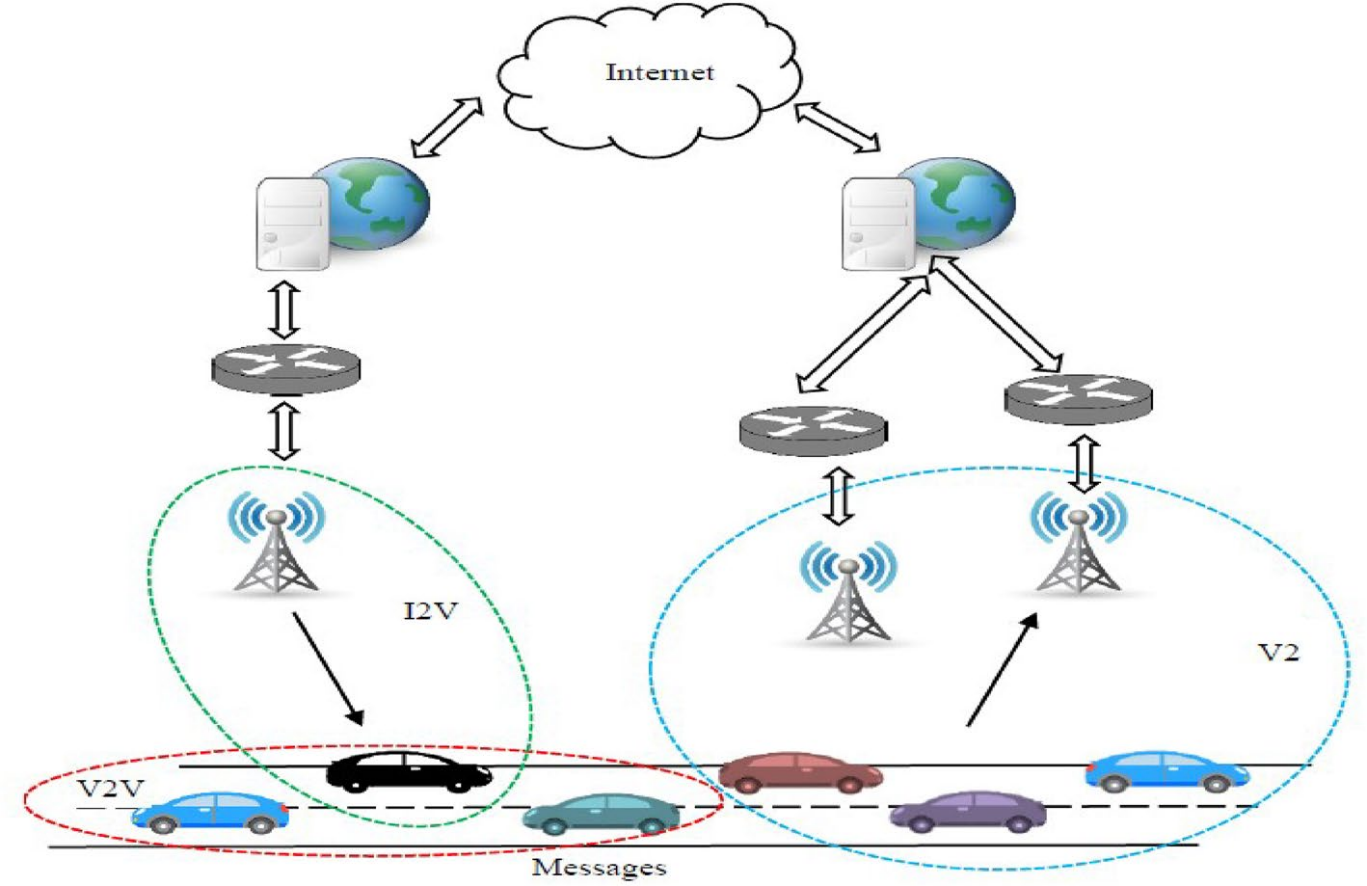
Architecture of a VANETs.

VANETs are categorized into three communication models comprises of Vehicle-to-Roadside (V2R) communication, Vehicle-to-Infrastructure (V2I) communication, and Vehicle-to-Vehicle (V2V) communication, as illustrated in Fig. 1.

V2V can provide a VU with information like collision detection^25^, emergency braking and traffic conditions. V2I can communicate the information to network infrastructures and vehicles^26^. The vehicle established a connection to RSUs in this domain for data exchange with other networks. Further, it requires a uninterrupted bandwidth to make it non-prone to attack due to communications with its infrastructure^27^.

Cooperative-Vehicle-to-Everything( C-V2X) technology was launched recently; a single platform that supports V2X communications^28^. It is a unique connectivity platform. C-V2X is a strong communication technology that is developed within the framework of the third-generation partnership project (3GPP)^29^. It connects each car and enhances traffic efficiency by enabling Cooperative Intelligent Transport Systems (C-ITS)^30^.

3GPP released its first V2X communications support in 2016 and the standards include Long-term Evolution (LTE)^31^. Due to their high data rate, high coverage, and penetration rate, LTE has robust advantages in V2I communication. However, the limited services and centralized structure to support V2V communication means LTE tends to face many challenges^32^. In addition, VANET communication is categorized into the following categories,

- **Warning propagation message** If a scenario is critical, the notification will be sent to a specific vehicle or set of vehicles^33^. A new routing algorithm is needed to address this problem and can be employed to forward warnings to the location^34^.
- **Vehicle beaconing** Beaconing is allowed to send beacon messages to RSUs and nearby vehicles, which includes the information on speed, acceleration and velocity^35^.
- **Infrastructure to vehicle warning** This type of messages is transmitted from RSUs to VU in order to improve traffic flow and road safety when possible crashes or crashes occur, particularly on a curve road, intersections or on narrow roads^36^.

### VANET characteristics

As a certain class of MANETs, VANETs have several features, e.g. they are both self-arranged and mobile node-composed networks. However, VANETs still have many special characteristics.

- **High computational capability:** In comparison with other mobile nodes (such as smart phones), operating vehicles can use significantly higher computational, communication and sensing capacity^37^.
- **Predictable mobility:** Vehicles are moving more predictably than typical MANET nodes. Only moving vehicles over roads. Positioning systems and map-based technologies can provide road information. In accordance with the speed and trajectory of a vehicle the future position can be estimated^38^. A determinant parameter for predicting vehicle mobility is also the hour of day or the specific day of the week .
- **Large scale:** A vehicle network, including numerous participants, could include an entire road network. Its coverage can extend from the district to the whole city. A VANET can reach tens of kilometers easily on highways^39^.
- **High mobility:** Vehicles topology in a VANET is highly dynamic and has different configurations^40^. For example, the topology of a vehicle leaving an avenue to go to a residential area may change dramatically. The density of nodes is an important factor in this regard. If the vehicle density is very high, as in early hours, change in topology may be minimal. On the other hand, very low vehicle density, for example during weekdays, is causing more topological changes due to high mobility^41^.
- **Partitioned network:** Due to the nature of traffic, vehicle networks are often divided. There are inter-vehicle gaps in residential and rural areas, since these scenarios are dwindling. This consists of a number of isolated node clusters^42^.

### Technical issues in VANET

However, a number of factors that have a critical impact on VANET targets have to be taken into account, so that the key problems which need to be resolved are specified. The following are the major questions from a technical point of view.

- **Bandwidth limitations:** VANETs lack the central coordinating bandwidth management and restriction of containment operations for the regulation of communications in V2V / V2I^43^. The bandwidth frequency range is also limited and the Dedicated Short Range Communication (DSRC) allocation of 75 MHz for VANET applications is limited, so that channel congestion can easily occur in high-density environments. Of course, bandwidth utilization should be optimized efficiently, which a major impact on routing has based on Quality of Service (QoS). For example, when the wireless channel is busy, a vehicle that needs packet transfers must wait for the unfair statement of this channel for some time so that the time the packet is transmitted increases especially in high-traffic scenarios^44^.
- **Connectivity:** In VANETs, network fragmentation often takes place because of the high mobility of vehicles and the rapid changes in topology, thus, it is better to extend the duration of the linking communication as long as possible^45^. This task can be achieved by increasing transmission power, but at the expense of severe interference and deterioration of the performance. Connectivity is therefore regarded in VANETs as an important matter. Although many MANET studies have been carried out in order to solve this problem, there are still many efforts being undertaken in order to develop VANETs^46^.
- **Small effective diameter:** The QoS between two nodes is degraded due to the small efficient network diameter of the VANETs, and the retention of a global system topology is impossible to achieve for one node. The limited effective diameter makes it difficult to directly use existing routing algorithms in VANETs^47^.
- **Routing protocol:** Due to complex movement of vehicles and highly diverse topological modifications^48^, it is a critical challenge for VANETs to design an efficient routing protocol that can successfully deliver a packet with the shortest possible time. This paper focus mainly on the problems associated with routing protocols.

### Critical challenges in routing

There are several routing techniques exist for assigning unique logical addresses to vehicles. However, the currently available routing protocols do not guarantee the prevention of duplicate address allocation within vehicle networks. The VANET encounters several challenges, including configuration issues, demographic factors, the quantity of vehicles, mobility patterns, and the unpredictable movement of vehicles that join and leave the network, as well as road dimensions that fall short of transmission coverage^49^.

The primary objective of the routing algorithm is to identify and sustain the most efficient route for transmitting data packets through intermediary nodes. The operation of VANETs is intricate due to the mobile nodes’ dynamic nature, which necessitates the utilization of specialized routing protocols from MANETs. Topology-based routing protocols require a distinct address for each participating node. Consequently, a mechanism is necessary to allocate distinct addresses; however, these protocols do not ensure the absence of address assignment duplication within the network. Hence, it can be inferred that the MANET algorithms are not appropriate for VANETs. Furthermore, the conventional routing protocols are inadequate in addressing certain VANET concerns, including but not limited to network topology, mobility model, traffic volume, rapid changes, and road width.

In the design of adhoc inherent networks, routing protocols typically adopt two distinct approaches: routing table-driven and source-initiated routing procedures on demand. Table-driven routing, also referred to as proactive routing protocols, is responsible for maintaining routing information. This facilitates the connection of each Virtual User (VU) to all other VUs within the network. The aforementioned protocols facilitate the dispensation of periodic updates by each node while maintaining a lucid and uniform perspective of the network topology. Proactive routing protocols offer the advantage of maintaining the destination route in the background, thereby avoiding low latency in real-time scenarios. This, in turn, prevents the occurrence of unused data paths that can potentially diminish the available bandwidth.

### Problem statement

The implementation of an efficient routeing protocol tailored to vehicle density is of utmost importance in the context of Vehicular Ad-hoc Networks (VANETs), given the plethora of routeing protocols available. The matter at hand pertains to the incidence of said detection. Upon the discovery of the density data, the aforementioned information is disseminated among other vehicles, resulting in an increase in central command. Moreover, the duration required for convergence escalates as the vehicular density experiences frequent fluctuations at a heightened pace.

The occurrence of inaccurate data in actual time has a detrimental effect on the operational efficiency of the routing protocol. The optimal local problem arises when vehicles solely rely on information from their next or subsequent road segment, as the routing protocol involves a per-hop computation.

The utilisation of machine learning models can be considered as a viable approach to facilitate the route selection process in Vehicular Ad-hoc Networks (VANETs). The implementation of machine learning techniques can aid Roadside Units (RSUs) in mitigating traffic congestion and regulating vehicular mobility. It is feasible to ascertain the level of risk posed by automobiles within their respective communication radius. This ensures that the packet is efficiently routed through accurate predictions along optimal pathways. The machine learning model predicts the state of the vehicle in real-time by analysing both current and past vehicle conditions. This type of prediction provides insights into the optimal routeing protocol in terms of efficiency.

### Motivation of the work

VANETs are becoming a key research motivator for governments, automobile manufacturers, and academic institutions due to their wide range of applications, high efficacy, and limited infrastructures, and attracting an increasing number of their demands. However, effective VANET implementation requires a number of essential aspects, one of which is how to construct adaptive and efficient routing pathways from origin to endpoints in complicated and congested urban environments. The benefits of VANETs will be restricted unless this routing challenge is solved.

The major contributions of the paper as follows,

- To use a IDRL technique for the purpose of routing the VU in VANETs by eliminating the problems of increasing control overhead associated with non-optimal communication between the VUs.
- To select the optimal routing path and to reduce the control overhead and longer convergence time using a machine learning algorithm in high dense VANETs.
- To monitor the behavior of the road segment, analyze and forecast the transmission capacities and availability of the vehicles using the IDRL technique. This technique allows RSU to maintain traffic information on IDRL-used roads to enhance network capacity performance. Furthermore, the IDRL forecasts the time to transmit based on the VU location. This is done by RSU to find the neighboring vehicle to transport the packages in real time.

## Literature review

Vehicle routing in VANET is an ever-growing challenge that seeks to identify the most efficient collection of routes for a given fleet of vehicles, although numerous efficient-routing protocols have been devised to tackle these concerns. This section presents the brief outline of the past researchers to handle the routing techniques in VANETs and about how well the author(s) tried to overcome the problems associated with routing techniques

Wu et al.^50^ proposed a Q-learning fuzzy constraint algorithm to implement an Adhoc On-demand Distance Vector (AODV) routing protocol employs a coherent methodology to assess the quality of wireless connections by considering multiple metrics, including bandwidth, link quality, and vehicular mobility.

According to Doolan & Muntean^51^ Time-Ants, it is posited that a specific amount of pheromone or traffic is allocated to each vehicle at any given time of the day. The vehicle’s road selection is determined in a timely manner through the use of an innovative algorithm based on traffic ratings. After multiple iterations, an optimal global traffic system is achieved. The utilisation of machine learning techniques enables the detection and mitigation of bottlenecks to gauge the density of traffic.

By Sanguesa et al.^52^ the V2X-d architecture was introduced. V2X-d utilises the integration of V2I (Vehicle-to-Infrastructure) and V2V (Vehicle-to-Vehicle) communication. The system acquires data on RSUs and vehicle characteristics through sensor technology, and employs road mapping techniques to precisely gauge vehicular density in real-time. Dynamic anchor based V2V protocols adopts routing under two scenarios which is examined by Jabbarpour et al.^53^.

The Machine learning assisted path selection (MARS) was proposed by Lai et al. (2016) as a means of evaluating the requisite data for routing protocols^54^. The maintenance of road data in MARS is facilitated through the utilisation of machine learning techniques within roadside units.

In their study, Dey et al.^55^ evaluated the efficacy of a Het-Net comprising diverse wireless V2V communication technologies. The method of handoff at the application layer facilitates the acquisition and transmission of collision warning data in Het-Net communication. The research demonstrates that Het-Net enhances V2V (vehicle-to-vehicle) communications. However, the study posits that the utilisation of Het-Nets has resulted in a compromise of application performance in the realm of traffic data collection. Unlike safety applications for vehicles, lower latency is not a requirement. In their study, Zhao et al.^56^ proposed an algorithm aimed at enhancing the efficacy of vehicle data, specifically through the utilisation of Support Vector Machine (SVM), by processing and generating routeing metrics. This approach examines the analytical and processing techniques utilised for analysing automobile data, while also exploring the potential implementation of machine learning algorithms in the context of VANET routing. Shu et al.^57^ introduced a method for Quality of Service (QoS) data dissemination that enhances the efficiency of data and QoS dissemination in a hierarchical Vehicular Ad-hoc Network (VANET). The proposed approach employs an improved Kruskal algorithm. The proposed methodology entails the utilisation of Kruskal algorithm to generate trees with minimal stretching in each road segment. The car units were grouped through the intra-cluster QoS technique, employing the c-mean clustering method. The designated cluster head of each spanning tree is responsible for gathering data from the leaf nodes and distributing it to other coordinator nodes, as well as performing the reverse operation. Tang et al.^58^ have put forth a proposal for a centralised routeing system in VANET, which incorporates mobility prediction. This system is supported by a network controller that is based on artificial intelligence and is powered by software. Advanced artificial neural network technology can facilitate precise mobility prediction by the Software Defined Network (SDN) controller. The Software-Defined Networking (SDN) controller gathers network information from Roadside Units (RSUs) and Base Stations (BSs), which are regarded as switches. The Software-Defined Networking (SDN) controller utilises global network information to compute the most efficient routeing paths for switches. The RSUs and BS will make independent decisions regarding routeing with the aim of minimising the total time spent on vehicle servicing.

## Proposed model

VANETs comprise a multitude of vehicles, each possessing a distinct and individualised identity. RSUs are present alongside the roadways and are linked to the Internet through a service provider. The proposed system under consideration involves V2I communication, which comprises of either a single Access Point (AP) or a single RSU within a designated geographical region. RSUs regularly transmit or receive vehicle data to and from the default portal, which is then relayed to the network operator and subsequently disseminated to system users.

**Figure 2:**
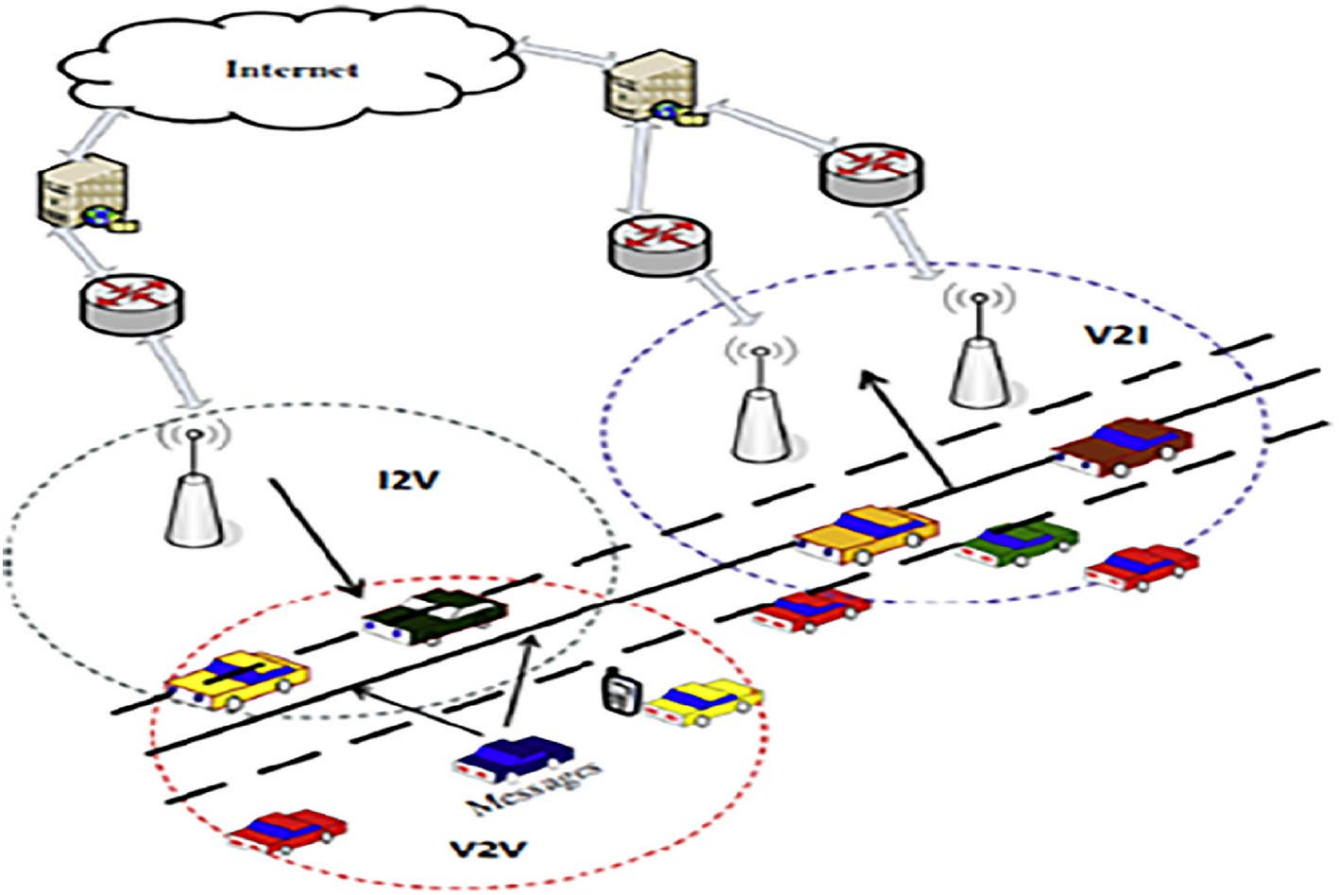
Routing technique of Proposed model.

The current techniques lack the inclusion of V2I cluster-based communication. The process involves segmenting the road into clusters and determining the traffic density and vehicle speed, as illustrated in Fig. 2. It is widely believed that as the number of vehicles on the road increases, there is a corresponding increase in traffic congestion, resulting in a decrease in the average speed of vehicles.

Let *l* be the current location of all the VUs $l = \{l_1, l_2,...,l_n\}$, the distance between the adjacent RSUs are $d = \{d_1, d_2,...,d_n\}$, set of routes are $r = \{r_1, r_2,...,r_n\}$ and the set of vehicle density is given by $V_d = \{V_{d_1}, V_{d_2},...,V_{d_n}\}$. The transition function takes the transition from one state to the next into account:

1 $$\begin{aligned} \Omega _{pq}: \phi \rightarrow r \;\; where \;\; 1 \le p \le n, 1 \le q \le n \end{aligned}$$

The function $\Omega _{pq}$ serves as a probabilistic mechanism to ensure that the feasible paths are present on the right-hand side of Eq. (1). The system in question typically functions in accordance with the input parameter $\phi = (l,d,V_d)$. The estimation of the distance between the vehicle and RSU is conducted by determining the current location of the vehicle and the vehicle density. This information is then utilised to derive the most efficient routes for forthcoming vehicles.

By calculating the total vehicles in a specific area, the vehicle density is estimated on all VUs, which is accessible over entire VANETs at a particular instant. This is estimated using Eq. (2),

2 $$\begin{aligned} V_d = \frac{\sum _{p=1}^{q} \gamma _p}{\sum _{p=1}^{q} \eta _p} \end{aligned}$$

where $\gamma _p$ - total VU in the region *i* and $\eta _p$ - *t* time of all vehicles.

The communication’s maximum coverage distance is 150 metres, and the thresholds are predetermined. Each geographical region exhibits a distinct vehicular density. Thus, it is imperative to perform accurate mapping, as outlined in Eq. (3), to map the routes by utilising the Transition Probability Matrix (TPM) values of the existing route. The TPM values possess the capability to select optimal pathways for the distribution of information.

3 $$\begin{aligned} \left[ {\begin{array}{cccc} \Omega _{11} &{} \Omega _{12} &{} \cdots &{} \Omega _{1n} \\ \Omega _{21} &{} \Omega _{22} &{} \cdots &{} \Omega _{2n} \\ \vdots &{} \vdots &{} \ddots &{} \vdots \\ \Omega _{n1} &{} \Omega _{n2} &{} \cdots &{} \Omega _{nn}\\ \end{array} } \right] \end{aligned}$$

The approach proposed is to find the best route for this particular problem, which is laid out with a minimum parametric weight.

### IDRL routing protocol

The proposed technique does not employ a predetermined routing protocol. The IDRL model is utilised to forecast the variation in $V_d$, which is subsequently extrapolated to predict vehicular motion on roadways. The selection of routes is dynamically determined based on the transmission capacity and a high probability of success upon reaching the IDRL driver. The system undergoes training through the utilisation of vehicle speed and movement in relation to the nearby RSUs that the vehicle unit traverses. The data undergo dynamic updates in response to the proximity of the incoming unit to the RSU.

The RSUs are interconnected via wired lines to facilitate the transmission of entry vehicle data within the study coverage area. This facilitates the dissemination of information to adjacent Roadside Units (RSUs) pertaining to the arrival, specifically with regards to its position and velocity. The determination of the position of vehicle nodes using GPS is challenging due to the high velocity at which the vehicles move. The hop-by-hop packet transmission methodology poses a challenge for GPS tracking of the vehicle. The utilisation of GPS positioning technology gives rise to concerns regarding security and privacy. The proposed approach employs deep reinforcement learning to mitigate the challenge of locating a vehicle’s position.

#### Route establishment phase (REP)

The process of transmitting packets to nearby VUs commences with the REP phase. The packages comprise distinct attributes, namely the present location of the vehicle, proximity to the surrounding area, density of vehicles, and the current itinerary. The intermediate node provides pertinent information, including its present location, network density, and latency within a specific area. The selection of intermediate nodes can be optimised by utilising an intelligent system to determine the optimal path for traversing the hops towards the target node.

#### Optimal route establishment (ORE)

The packet pertaining to the vehicle unit comprises of several parameters, such as the distance of the vehicle unit from RSUs, the real-time position of the vehicle, the end-to-end delay, and the varying density of vehicles.

The process of establishing an optimal route by the IDRL involves a series of steps that rely on the application of rewards or penalties. The establishment of a source and destination routing path is facilitated by a transition function ($\Omega _{pq}$) that enables the transition between various phases. The modification of $\Omega _{pq}$ values is contingent upon the action taken, specifically, the alteration of the path threshold. The transportation network’s capacity to transmit data packets is contingent upon the road limit, which is determined by the number of tracks available for routeing from the origin to the destination, as well as the combined weight of these tracks. Route weights and total weights are present on the routes. These factors facilitate the identification of appropriate actions, whereby a routeing path is either penalised or rewarded.In the event that the present node is considered to be the intended node, the weights are consistently revised and the accumulated weights, denoted as $\mu$ and constrained within the range of [0, 1], are determined by the reward function. In cases where the present node does not correspond to the intended target node, the cumulative weights are modified through the utilisation of the transition function. A constant function can be employed to assess the reliability or efficacy of each route. The rate of convergence towards optimal pathways is expected to be higher in instances where the value of $\mu$ approximates 1. If the value of $\mu$ approaches zero, the attainment of an optimal routeing path will be postponed.Conversely, in cases where transmission proves to be inefficient, the total weight is reduced by a value of $\mu$ $\in$ [0, 1]. The IDRL is subject to penalties that are mitigated in cases of communication failure, thereby reducing or altogether preventing its penalisation. The nodes of the vehicle have the capability to decline an incoming packet. Furthermore, their matrices of activity likelihood are periodically revised, taking into account the response and routing path thresholds.

#### Route selection phase

Following the initial routing step, the next action is to choose the best neighbor node from the routing list. It makes use of a routing protocol in particular. In this case, each vehicle or node acquires the optimum phenomenological value of the nearby vehicle for communication across the system, based on the neighborhood and vehicle density of the forwarding messages.

The period is separated into sub-phases with various interval times, where the timing interval is equal and the vehicle intensity is approximated based on vehicle density. The vehicle’s speed and density tend to fluctuate owing to traffic during the next interval. As a result, it is important to assess the traffic density at a certain time period using a routing algorithm in order to pick the appropriate track.

#### Route selection phase using IDRL

Based on the previous observations the IDRL route finds the optimal routing paths. The IDRL that predicts the next TPM is used to compute optimal routes efficiently. This can be considered a supervised problem of learning.

## Supervised learning

The current study focuses on a supervised learning task that involves a sample and a labelling space, denoted as *P* and *Q*, respectively. The current study focuses on the supervised learning task of IDRL (*X*), wherein a function is established to map values in *P* to their corresponding labels in *Q*. The purpose of the mapping function is to establish a correspondence between a sample set ($p_i$) and its corresponding true labels ($q_i$), both of which belong to the Cartesian product of *P* and *Q*. The process of mapping generates accurate designations for the novel set of samples, which are acquired through a distribution that is akin to the data.

The proposed system employs an IDRL mechanism that effectively monitors the history of TPM across various iterations, spanning from prior to the current iteration to the present. This model forecasts the optimal routes for transmitting TPM-anticipated packets. The generation of TPM sequences is contingent upon the present and historical TPM of the system under consideration. The evaluation of the protocol is often deemed challenging due to the perceived unpredictability and significant skewness of traffic pattern generation, which is based on a probability distribution.By utilising the knowledge and skills of prospective TPMs and implementing an effective mapping approach, the IDRL is capable of predicting the most efficient routes for forthcoming roadways. The IDRLs acquire knowledge from the observed TPM through the mapping strategy in a direct manner. The agent engages in iterative interactions with the vehicle environment within the framework of Inverse Reinforcement Learning (IDRL). The temporal and spatial components of the road network are partitioned into discrete time intervals (*t*) and sub-sections..

## Results and discussion

The evaluation of the proposed method’s performance was conducted utilising the Network Simulator (NS-2.35) simulation tool. The simulation is conducted within an area measuring 1000 metres by 1000 metres. VanetMobiSim is a software tool that models the mobility patterns of automobiles and their corresponding actions in relation to the urban setting. The RSUs exhibit a stochastic spatial pattern, characterised by a diverse range of velocities spanning from 5 to 30 m/s for individual vehicles. The transmission power is adjusted to achieve the desired range, with the upper limit of the transmission range being 250 metres. The data packets have a fixed size of 512 bytes and are transmitted from the source node using the Constant Bit Rate (CBR) protocol.

The present study involves a comparative analysis of the proposed technique vis-à-vis Adaptive Ranking based Energy efficient Opportunistic Routeing (AREOR)^5^, Improved-AREOR (I-AREOR)^6^, and Adaptive Ranking based Improved Opportunistic Routeing (ARIOR)^7^. The IDRL routeing technique is subjected to a comparative analysis with existing peer routeing techniques, namely AREOR, I-AREOR, and ARIOR, for the purpose of evaluating Packet Delivery Ratio (PDR). The RSU coverage ratio is a defined metric that expresses the proportion of RSU coverage in relation to the total area. The IDRL determines the transmission and destination position capabilities through the utilisation of RSU data.

**Figure 3:**
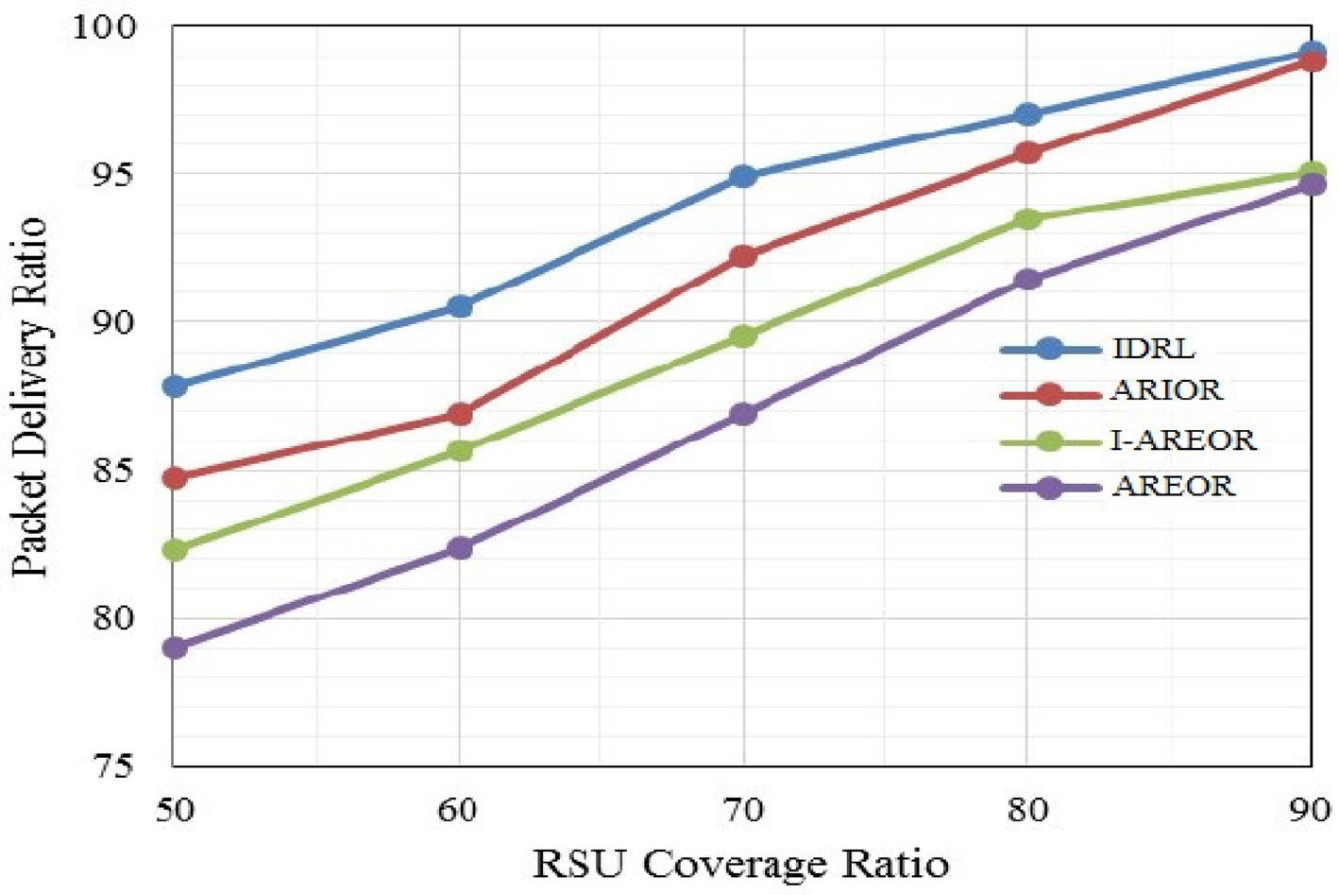
Delivery ratio (%) Vs. coverage ratio (%).

The data presented in Fig. 3 indicates a notable enhancement in the coverage area for transmission. The data indicates that the network’s performance exhibits an upward trend across all four methodologies as the coverage range of the Roadside Units (RSUs) expands. The proposed process has been observed to attain a higher Packet Delivery Ratio (PDR) compared to existing routeing techniques employed by peers. The method under consideration has demonstrated a noteworthy enhancement, with an average increase of 5% in comparison to the prior ARIOR. This improvement can be attributed to the retention of connectivity information between units through the utilisation of announcement messages in the IDRL approach.

**Figure 4:**
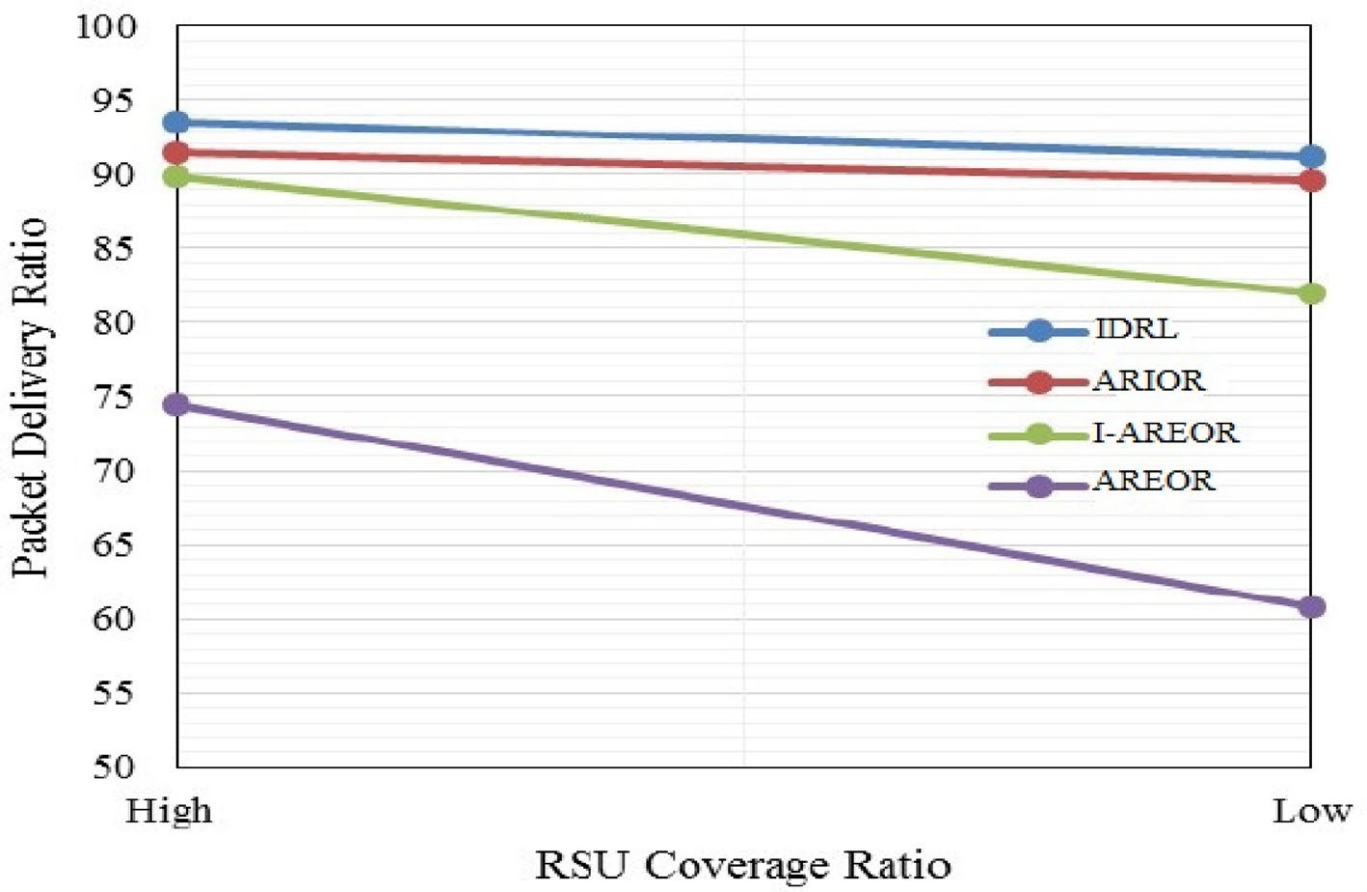
Delivery ratio (%) Vs. control overhead.

Figure 4 illustrates the comparison among PDR and vehicles with low and high densities. The proposed method exhibits a 6.5% increase in performance compared to the currently employed ARIOR method. Alternative approaches exhibit instability due to their reliance on distributed architecture, which leads to heightened density. The findings indicate that the utilisation of RSUs leads to a 23.

**Figure 5:**
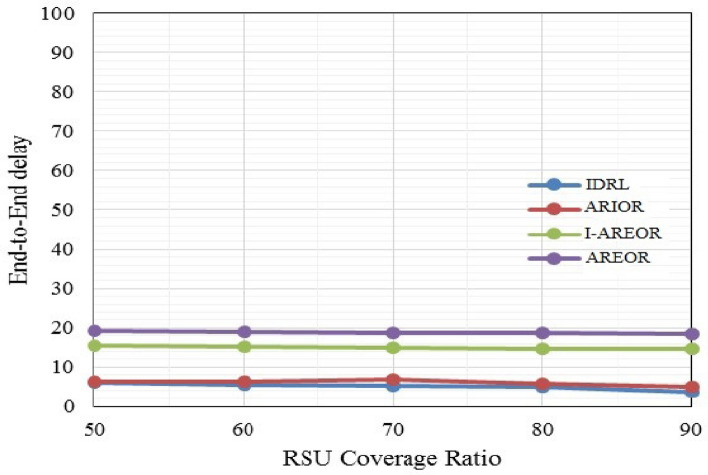
End-to-End delay (s) Vs. coverage ratio (%).

**Figure 6:**
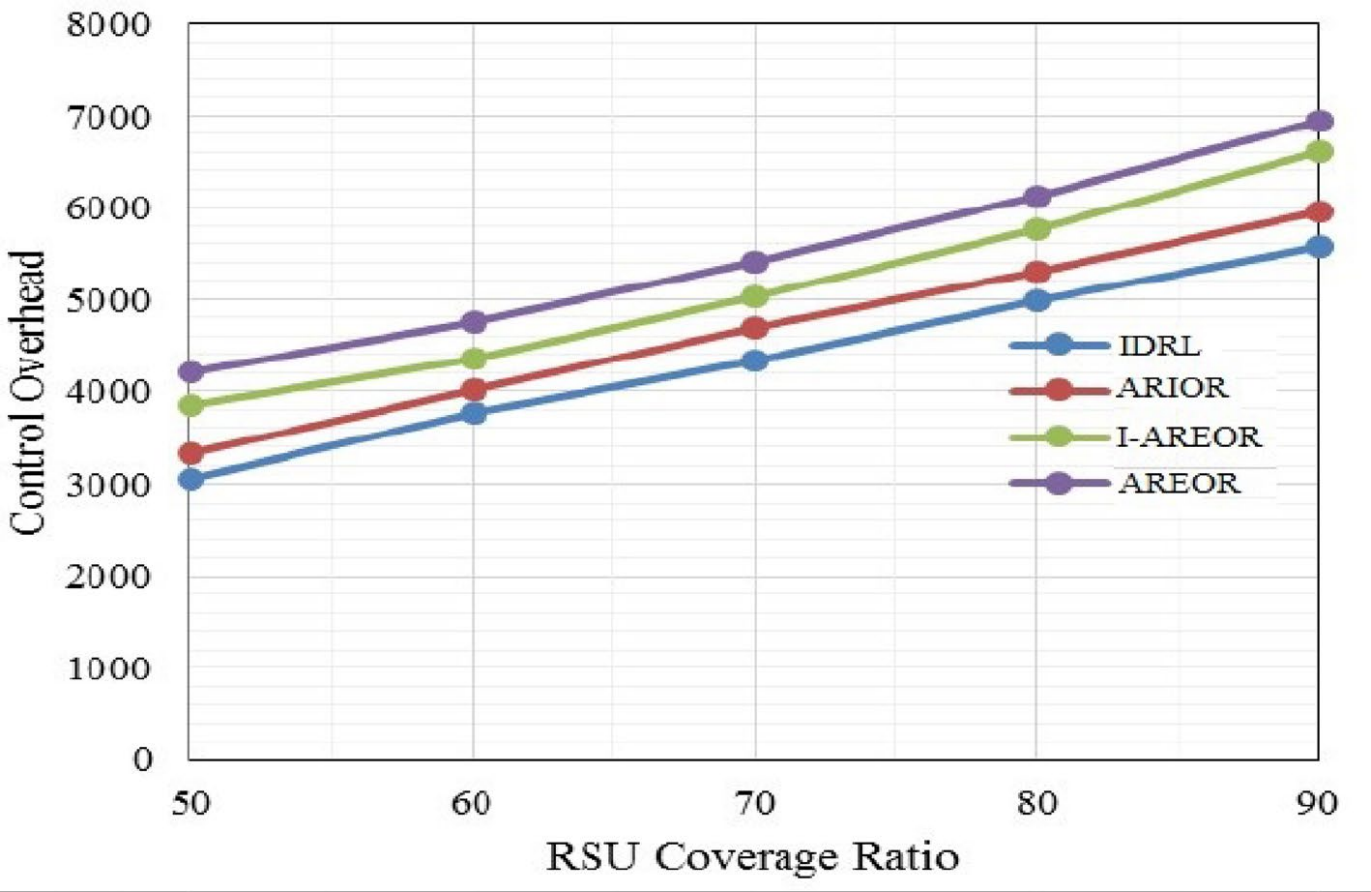
Control overhead (per min) Vs. coverage ratio (%).

The outcomes of the final delay are depicted in Fig. 5. The findings indicate that the inter-RSU packet forwarding delays are significantly influenced by the extent of RSU coverage. Significantly reducing the transmission area results in a substantial reduction in associated delays. The findings indicate that the resolution of RSU information remains consistent in the proposed methodology when compared to other routeing techniques currently in use. The comparative analysis of control overhead in VANETs between the AREOR, I-AREOR, ARIOR and the IDRL is presented in Fig. 6. The control overhead is responsible for specifying the data utilised in the training of the reporting units of the vehicle for the purpose of inter-vehicle communication with the Roadside Units (RSUs). The implementation of the IDRL has demonstrated a reduction in overhead controls when compared to existing methods. As the network density increases, the total number of RSUs is also increasing, leading to a rise in overhead. Nonetheless, the IDRL exhibits a diminished overhead control in contrast to analogous routeing methodologies currently in use, while maintaining a coverage ratio of 90% for both the IDRL and aforementioned routeing methodologies.

The IDRL technique exhibits a high potential for achieving favourable success rates. The proposed methodology has demonstrated greater precision in evaluating peer routeing techniques by utilising data collection pertaining to vehicle density, direction, and speed. In addition to extant peer routeing methodologies, data transmission occurs with a high degree of dependability. Finally, it can be observed that the employment of the IDRL route yields a superior Packet Delivery Ratio (PDR) coupled with reduced latency, as well as an optimised overhead control mechanism that amplifies the efficacy of data transmission. The simulation results indicate that the scale of IDRL routeing in VANETs is superior to that of AREOR, I-AREOR, and ARIOR.

## Conclusion and future scope

The present study underscores the difficulty in effectively managing, mitigating, and minimising the persistence of transmission. The IDRL technique proposed in this study places emphasis on the selection of roads with high traffic density in order to enhance packet transmission during the establishment of a route. The technology diminishes the duration of data transmission and autonomously monitors the density of traffic with heightened accuracy. The aforementioned process involves partitioning the entire area into multiple clusters and fine-tuning the pathway based on various input variables, including the density and positioning of the VU. The IDRL technique enhances the efficiency of the routing trajectory and minimises the time required for convergence in the context of varying vehicular densities. The IDRL effectively monitors, analyses, and predicts routeing behaviour by utilising transmission capacity and vehicle data. As a result, the reduction of transmission delay is achieved through the utilisation of adjacent vehicles for packet transportation in V2I communication. The findings of the simulation demonstrate the efficacy in relation to Packet Delivery Ratio (PDR), velocity of vehicles, density of vehicles, range of transmission, total count of Access Points (APs), and delay in network. The potential for future development lies in the incorporation of supplementary attributes such as anonymity, mutual authentication, and intractability, which would provide reduced communication and computational expenses in domains such as e-healthcare, intelligent transportation, and smart ecosystems.

## Data Availability

The datasets used during the current study are available from the corresponding author upon reasonable request.
